# Gene expression profiling of mesenteric lymph nodes from sheep with natural scrapie

**DOI:** 10.1186/1471-2164-15-59

**Published:** 2014-01-23

**Authors:** Hicham Filali, Inmaculada Martín-Burriel, Frank Harders, Luis Varona, Carlos Hedman, Diego R Mediano, Marta Monzón, Alex Bossers, Juan J Badiola, Rosa Bolea

**Affiliations:** 1 Centro de Investigación en Encefalopatías y Enfermedades Transmisibles Emergentes, Facultad de Veterinaria, Universidad de Zaragoza, Zaragoza, Spain; 2 Laboratorio de Genética Bioquímica (LAGENBIO), Facultad de Veterinaria, Universidad de Zaragoza, Zaragoza, Spain; 3 Central Veterinary Institute part of Wageningen UR (CVI), Lelystad, The Netherlands; 4 Unidad de Genética Cuantitativa y Mejora Animal, Facultad de Veterinaria, Universidad de Zaragoza, Zaragoza, Spain

**Keywords:** Natural scrapie, Mesenteric lymph node, Microarray, Gene expression, Real time PCR, Prion

## Abstract

**Background:**

Prion diseases are characterized by the accumulation of the pathogenic PrP^Sc^ protein, mainly in the brain and the lymphoreticular system. Although prions multiply/accumulate in the lymph nodes without any detectable pathology, transcriptional changes in this tissue may reflect biological processes that contribute to the molecular pathogenesis of prion diseases. Little is known about the molecular processes that occur in the lymphoreticular system in early and late stages of prion disease. We performed a microarray-based study to identify genes that are differentially expressed at different disease stages in the mesenteric lymph node of sheep naturally infected with scrapie. Oligo DNA microarrays were used to identify gene-expression profiles in the early/middle (preclinical) and late (clinical) stages of the disease.

**Results:**

In the clinical stage of the disease, we detected 105 genes that were differentially expressed (≥2-fold change in expression). Of these, 43 were upregulated and 62 downregulated as compared with age-matched negative controls. Fewer genes (50) were differentially expressed in the preclinical stage of the disease. Gene Ontology enrichment analysis revealed that the differentially expressed genes were largely associated with the following terms: glycoprotein, extracellular region, disulfide bond, cell cycle and extracellular matrix. Moreover, some of the annotated genes could be grouped into 3 specific signaling pathways: focal adhesion, PPAR signaling and ECM-receptor interaction. We discuss the relationship between the observed gene expression profiles and PrP^Sc^ deposition and the potential involvement in the pathogenesis of scrapie of 7 specific differentially expressed genes whose expression levels were confirmed by real time-PCR.

**Conclusions:**

The present findings identify new genes that may be involved in the pathogenesis of natural scrapie infection in the lymphoreticular system, and confirm previous reports describing scrapie-induced alterations in the expression of genes involved in protein misfolding, angiogenesis and the oxidative stress response. Further studies will be necessary to determine the role of these genes in prion replication, dissemination and in the response of the organism to this disease.

## Background

Transmissible spongiform encephalopathies (TSEs) or prion diseases are a group of fatal neurodegenerative disorders of animals and humans. TSEs generally involve long incubation periods, which in humans can span several decades [1]. These diseases are characterized by the accumulation, mainly in nervous and lymphoreticular tissues, of an abnormal isoform (PrP^Sc^) of a normal host-coded cell surface glycoprotein (PrP^c^). Pathogenesis typically involves a triad of histological events; vacuolation, neuronal loss and astrocytosis. PrP^Sc^ accumulation is currently considered the only reliable biochemical marker for this group of diseases [2].

Scrapie is a TSE that naturally affects sheep and goats. Ovine scrapie is a useful model in which to study the pathogenic mechanisms of the variant Creutzfeldt Jakob disease (vCJD), which affects humans [3]. Like vCJD, classical scrapie is associated with widespread tissue infectivity, mainly in the central nervous system (CNS), peripheral nervous system (PNS), and lymphoreticular system (LRS) [4].

Mesenteric lymph nodes are one of the first tissues of the LRS in which PrP^Sc^ accumulates during the preclinical disease stage in naturally infected sheep [5,6], although this process strongly depends on the animal’s *PRNP* genotype. Subsequently, other LRS tissues are rapidly and near simultaneously exposed to infectivity, presumably by circulating prions. In the clinical stages of the disease and in all susceptible *PRNP* genotypes except VRQ/ARR, infectivity and PrP^Sc^ accumulation are generally observed throughout all lymphoid tissues, except for the thymus [7]. Follicular dendritic cells (FDCs) may be necessary for prion propagation within the LRS [8], and macrophages and tingible body macrophages (TBMs) of the LRS have been identified as reservoirs of the infectious agents of TSEs [4,9].

The molecular mechanisms that underlie the uptake of the infectious prion agent and the progression of the disease remain largely unknown. Given the complexity and multifactorial nature of the spread and accumulation of the infectious agent, we and others have used gene expression analysis platforms to identify signaling pathways that are altered during PrP^Sc^ accumulation and subsequent neurodegeneration. This approach facilitates the identification of potential biomarkers and drug targets in natural ovine scrapie [10,11] and in experimental murine scrapie models [12-14].

Few gene expression-profiling studies have investigated changes in the lymphoreticular system in sheep with natural scrapie; most have been performed using murine models [15,16] or ileal Peyer’s patches from orally inoculated lambs [17]. In the present study, we used an ovine microarray technology to identify transcriptional changes in the mesenteric lymph node in both clinical and preclinical disease stages in sheep that were naturally infected with scrapie. This is the first transcriptome-wide expression profiling study of the lymphoreticular system in sheep with natural scrapie.

## Methods

### Ethics statement

This study was approved by the Ethics Committee for Animal Experiments of the University of Zaragoza (Permit Number: PI02/08) and was carried out in strict accordance with the recommendations for the care and use of experimental animals and in agreement with national law (R.D. 1201/2005).

### Animals, necropsy, tissue collection and RNA isolation

All details of the animals used and the necropsy and RNA isolation procedures have been previously reported [10,11]. Briefly, we used a 17 female ARQ/ARQ Rasa Aragonesa sheep (aged 1-6 years). Eleven animals were obtained from scrapie-infected flocks from several locations in Aragon (Spain); 7 of these exhibited clinical signs of scrapie while 4 were preclinical. Six animals were selected from flocks located in scrapie-free areas and were used as breed, genotype and age-matched controls.

Sheep in the clinical disease stage were identified by assessing clinical signs associated with the disease [18]. Third eyelid [18] and rectal mucosa biopsies [19] were used to confirm this diagnosis and to identify animals in the preclinical disease stage. Postmortem examinations revealed no additional pathological findings. Mesenteric lymph node samples were divided into 2 halves; one was snap-frozen in liquid nitrogen and stored at −80°C until RNA extraction, and the other was formalin-fixed and paraffin-embedded for further histopathological analysis. Total RNA isolation, purification and quality control were performed as previously described [10,11].

### Immunohistochemical detection of PrP^Sc^

Immunohistochemical (IHC) analyses were performed using serial sections. Prion protein was detected, after formic acid treatment and proteinase K digestion, using the monoclonal primary antibody L42 (1:500; R-Biopharm), as previously described [20]. Negative controls were performed omitting the primary antibody from control and scrapie sections.

The preparations were microscopically examined and global PrP^Sc^ deposition was scored by quantifying the proportion of lymphoid follicles with PrP^Sc^ deposits as follows: 1, <20%; 2, 20-50%; 3, >50% [19]. Significant differences between clinical, preclinical and control groups were identified using the Kruskal-Wallis test.

### Microarray hybridization

The custom CVI 4x44K microarrays used in this study contained custom eArray-designed 60-mer probes of previously sequenced, normalized and subtracted cDNA libraries of ovine Peyer’s Patch, obex and tonsil, supplemented with the publicly available *Ovis aries* transcripts from the NCBI/EBI databases and the Agilent *O. aries* transcript catalog. All arrays were printed using Sureprint technology (Agilent Technologies).

Preparation of the labeled cDNA probes and subsequent Genechip hybridizations were performed in accordance with the Agilent Technologies One-Color Microarray-Based Gene Expression Analysis guidelines, as previously described [10,11]. Hybridizations were performed in duplicate, resulting in 14 microarrays for the clinical samples, 8 microarrays for the preclinical samples and 12 for the negative control samples. Microarrays were scanned using the GenePix 4200AL Scanner (Axon Instruments) and GenePix Pro 6.0 software and the hybridization data were extracted using Agilent Feature Extraction software v9.5.3.1 (Agilent Technologies) before processing with GeneSpring GX 10.0.2 (Agilent Technologies). Chip values were normalized using the 75th percentile value and expression levels were calculated. Global mesenteric lymph node gene expression profiles for the clinical and preclinical disease stages were compared with the negative controls using a linear model that accounts for both technical (random animal effects) and biological replicates (disease effects). A multiple testing correction developed by Benjamini and Hochberg was also applied. Only genes that exhibited at least a 2-fold change (FC) in expression between healthy and infected animals and had a *P*-value ≤ 0.05 were selected. These genes were clustered according to their Euclidean distance coefficient using PermutMatrix software [21]. A Megablast search of the GenBank database nr/nt was performed to identify genes that were similar to the differentially expressed probes. The molecular functions of the genes were classified according to Gene Ontology (GO) using an on-line functional annotation tool (DAVID Bioinformatics Resources 6.7; NIAID/NIH, USA) [22,23].

### Quantitative real-time PCR

Quantitative real-time PCR (qRT-PCR) was performed to confirm the expression of 7 genes. Genes were selected based on previous studies describing their association with prion and other neurodegenerative diseases or their potential role in protein misfolding repair, the promotion of angiogenesis, or the systemic response of the organism to infection [24-30]. The PCR primer sequences used for the quantification of the genes encoding butyrobetaine (gamma), 2-oxoglutarate dioxygenase 1 (*BBOX1*), ceruloplasmin (ferroxidase) (*CP*), prefoldin subunit 2 (*PFDN2*), proteasome subunit, alpha type, 7 (*PSMA7*), serpin peptidase inhibitor, clade E (nexin, plasminogen activator inhibitor type 1), member 1 (*SERPINE1*), ubiquitin carboxyl-terminal esterase L1 (ubiquitin thiolesterase) (*UCHL1*) and vascular endothelial growth factor A (*VEGFA*) are shown in Table 1. The RNA samples used for qRT-PCR were the same as those used for microarray experiments. The qRT-PCR assays were designed using Primer Express 2.0 software (Applied Biosystems) to select appropriate primer sequences from known ovine or bovine sequences. Whenever possible, the exon-exon border was included to prevent amplification of genomic DNA in the PCR reaction. Complementary DNA (cDNA) was synthesized from 1 μg RNA using random hexamer primers with the Superscript First Standard Synthesis System for RT-PCR (Invitrogen). To confirm the elimination of any remaining DNA, reverse transcription was performed with and without the enzyme.

**Table 1:** Genes analyzed by quantitative real-time PCR

**Gene**	**Primer sequence**	**Size (bp)**	**Accession number**
*BBOX1*	F: TGCAAACAATGTGGCTTACACA	85	NM_001101881.2**
	R: AAGCTGAACCCCAGGTGGAT		
*CP*	F: GCAGCCAGATACTGCAGGGAC	97	NM_001009733.1*
	R: CCCGCACTGGCTCACAGTATAT		
*PFDN2*	F: GCAGGTAATTGCTGGCTTCAA	84	NM_001080221.2**
	R: TTCAACTCCATCTCCAGCTCAG		
*PSMA7*	F: TAATGTCTGCATGGCATTCGC	81	NM_001034233.2**
	R: TGGCTCTGGCATTCCACC		
*SERPINE1*	F: TGTACGTGTCGCAGGCGC	83	NM_174137.2**
	R: TACAAGGGCTGTGGAGGAGGAC		
*UCHL1*	F: AACTTGATGGACGGATGCCTT	84	NM_001046172.2**
	R: TGCAGACCTTGGCAGCGT		
*VEGFA*	F: GGGCTGCTGTAATGACGAAAGT	81	NM_001025110.1*
	R: GGTTTGATCCGCATAATCTGCA		

*Ovine cDNA.

**Bovine cDNA.

Primers (F, forward; R, reverse) used for gene amplification, amplicon size and GenBank accession numbers for the bovine cDNA sequences used for primer design.

Quantitative RT-PCR was performed using SYBR® Green (PE Applied Biosystems) assays as previously described [10,11]. To improve the normalization accuracy, a normalization factor (NF) was used to determine the expression level of each gene in each sample, as previously described [31]. The NF was calculated using the geometric mean expression of 3 housekeeping genes (GAPDH, G6PDH and RPL32). The primers and PCR conditions used for the amplification of these housekeeping genes have been described previously [31,32].

The quantitative results obtained from the qRT-PCR assays were expressed as FC with respect to controls. Significant differences between groups were determined using a Student’s *t*-test (*P* < 0.05).

### The relationship between neuropathology and gene expression

We used 2 different models to quantify the relationships between the PrP^Sc^ deposition and gene expression. We first used a simple regression model,

$$y_{i}=\mu +bp_{i}+e_{i}$$

where y_i_ is the gene expression data for the i^th^ individual for each gene, μ is the general mean, b is slope of the regression analysis, p_i_ is the PrP^Sc^ deposition for the i^th^ individual and e_i_ is the residual. In addition, we used another model,

$$y_{\mathrm{ij}}=\mu +T_{i}+bp_{\mathrm{ij}}+e_{\mathrm{ij}}$$

which includes a systematic effect associated with the 3 categories (Preclinical, Clinical and Healthy). Here y_ij_ represents the gene expression for the j^th^ individual at the i^th^ category, T_i_ the effects of the i^th^ treatment, p_ij_ the PrP^Sc^ deposition and e_ij_ the residual. The statistical significance of the slope associated with PrP^Sc^ deposition was determined for both models. Analyses were performed using the R project for Statistical Computing.

## Results

### PrP^Sc^ deposition in lymphoid tissue

**Figure 1:**
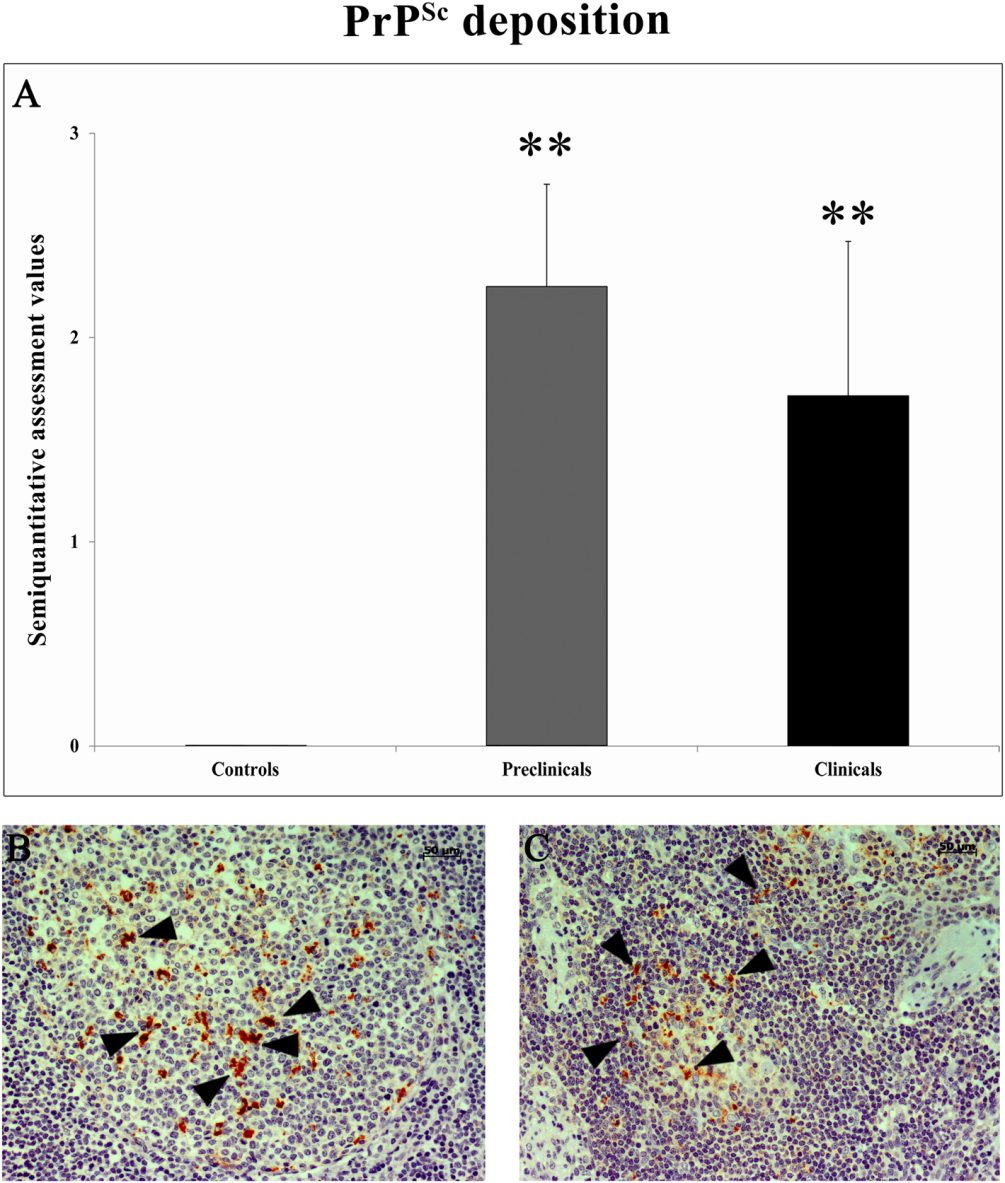
**Semi-quantitative assessment of PrP**^**Sc **^**deposition in mesenteric lymph nodes samples from control, preclinical and clinical scrapie sheep.** Deposition was evaluated on a scale of 0 (negative) to 5 (maximum staining intensity). Significant differences were detected between control and scrapie groups using a Kruskal-Wallis test (**P < 0.01) **(A)**. PrP^Sc^ deposition in the mesenteric lymph node from preclinical **(B)** and clinical **(C)** scrapie sheep.

The accumulation of PrP^Sc^ in the LRS was consistent with the features of classical scrapie. PrP^Sc^ was detected within the primary and secondary lymphoid follicles of the lymph nodes. Some cells in the follicular mantle and paracortical area were also PrP^Sc^-positive. Interfollicular and sinusal cells were rarely labeled. As seen in other LRS tissues, distinct granular immunolabeling was detected in the cytoplasm of tingible body macrophages in both the dark and light zones of the lymphoid follicles. A granular deposition was observed in the vicinity of round cells, which were morphologically similar to immature B lymphocytes (Figure 1). None of the control animals displayed PrP^Sc^ immunostaining and no statistically significant differences were observed between the 2 scrapie-infected groups (preclinical and clinical).

### Identification of differentially expressed genes in the mesenteric lymph node of sheep with natural scrapie

A total of 234 probe sets displayed significant differences in expression of 2-fold or more between the control and scrapie (preclinical and clinical) groups. While the genes of *Ovis aries* are relatively poorly annotated, we used BLAST searches against publicly available databases to identify a set of 177 known genes from the complete set of 234 differentially expressed probes. Some of these 234 probes encoded the same gene. The microarray data were deposited in ArrayExpress and assigned the accession number E-MTAB-1346.

**Figure 2:**
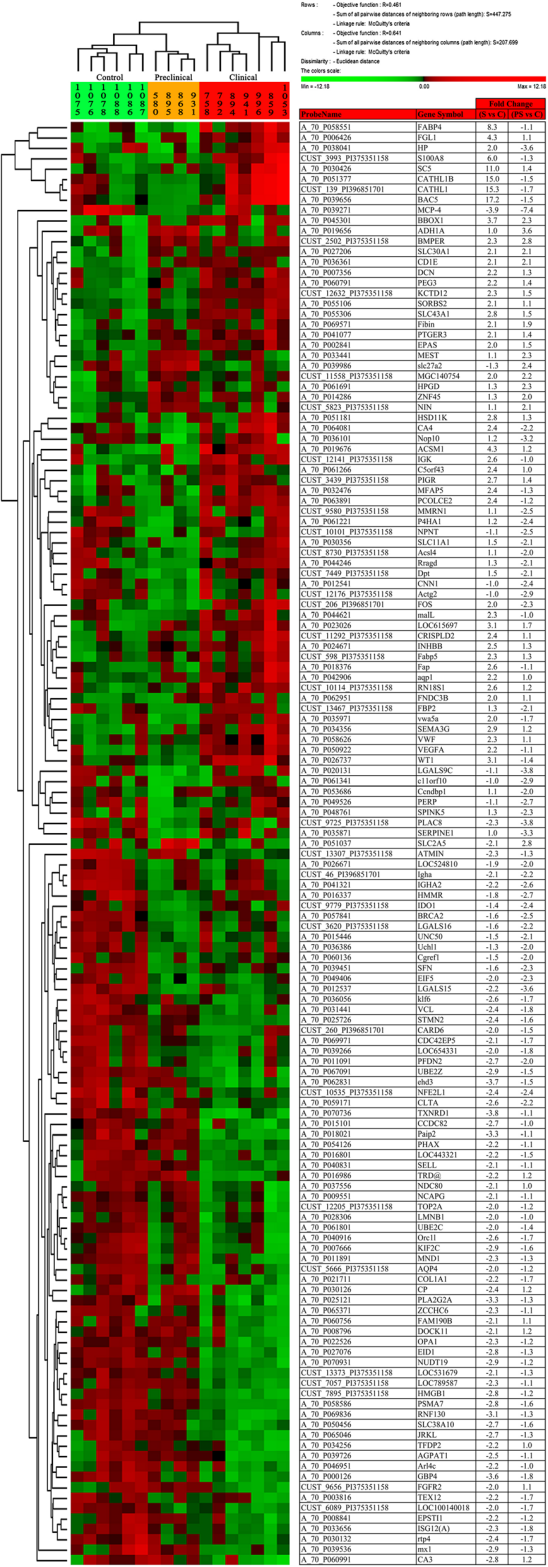
**Condition trees generated by cluster analysis.** A hierarchical cluster analysis (Euclidean distance clustering algorithm) was performed using PermutMatrix [21], which identified 139 genes whose expression differed significantly (≥ 2 fold change with respect to controls) in at least at one of the 2 disease stages analyzed (preclinical and clinical). Colors represent the level of expression. Sample information is listed across the top. The names of the known genes are indicated. Note the distinct patterns of gene expression in control, preclinical and clinical animals.

Based on the GO analysis of human homologs, 139 genes had known functions (Table 2), of which 105 were differentially regulated in clinical scrapie-infected sheep (43 upregulated and 62 downregulated) and 50 in preclinical scrapie-infected sheep (12 upregulated and 38 downregulated). The functional groups with the highest *p* values in the enrichment analysis are shown in Table 2; the most relevant terms were glycoprotein, extracellular region, disulfide bond and cell cycle. In addition, some of the annotated genes were grouped in 3 signaling pathways: focal adhesion, PPAR signaling pathway and ECM-receptor interaction (Table 3). After hierarchical clustering using the expression levels of all annotated genes, we were able to discriminate the 3 experimental groups: clinical, preclinical and healthy (Figure 2).

**Table 2:** GO terms for differentially expressed genes

**Term**	***P *** **value**	**ProbeName**	**FC (C vs NC)**	**FC (PC vs NC)**	**Gene symbol**	**Gene name**	**Citation**
Glycoprotein	5.16E-04	A_70_P042906	2.22	1.00	AQP1	Aquaporin 1 (Colton blood group)	
		CUST_5666_PI375351158	−2.02	−1.16	AQP4	Aquaporin 4	[10]
		A_70_P041077	2.09	1.39	PTGER3	Prostaglandin E receptor 3 (subtype EP3)	
		A_70_P030356	1.48	−2.06	SLC11A1	Solute carrier family 11 (proton-coupled divalent metal ion transporters), member 1	
		A_70_P051037	−2.09	2.85	SLC2A5	Solute carrier family 2 (facilitated glucose/fructose transporter), member 5	[10]
		A_70_P036386	−1.29	−2.01	UCHL1*	Ubiquitin carboxyl-terminal esterase L1 (ubiquitin thiolesterase)	
Extracellular region	5.37E-04	CUST_2502_PI375351158	2.26	2.76	BMPER	BMP binding endothelial regulator	
		A_70_P006426	4.26	1.06	FGL1	Fibrinogen-like 1	
		A_70_P069571	2.05	1.93	FIBIN	Fin bud initiation factor homolog (zebrafish)	
		A_70_P038041	1.95	−3.63	HP	Haptoglobin	[10]
		CUST_3439_PI375351158	2.70	1.38	PIGR	Polymeric immunoglobulin receptor	
		A_70_P035871	1.03	−3.29	SERPINE1*	Serpin peptidase inhibitor, clade E (nexin, plasminogen activator inhibitor type 1), member 1	
		A_70_P039451	−1.57	−2.28	SFN	Stratifin	
		A_70_P050922	2.19	−1.06	VEGFA*	Vascular endothelial growth factor A	
Disulfide bond	1.10E-03	CUST_598_PI375351158	2.28	1.33	FABP5	Fatty acid binding protein 5 (psoriasis-associated)	
		A_70_P070736	−3.83	−1.06	TXNRD1	Thioredoxin reductase 1	
Cell cycle	1.17E-03	A_70_P058586	−2.85	−1.55	PSMA7*	Proteasome (prosome, macropain) subunit, alpha type, 7	
		A_70_P057841	−1.61	−2.51	BRCA2	Breast cancer 2, early onset	
		A_70_P053686	1.11	−2.01	CCNDBP1	Cyclin D-type binding-protein 1	
		A_70_P060136	−1.48	−2.04	CGREF1	Cell growth regulator with EF-hand domain 1	
		A_70_P027076	−2.79	−1.28	EID1	EP300 interacting inhibitor of differentiation 1	
		A_70_P007666	−2.87	−1.61	KIF2C	Kinesin family member 2C	
		A_70_P011891	−2.34	−1.29	MND1	Meiotic nuclear divisions 1 homolog (S. cerevisiae)	
		A_70_P009551	−2.13	−1.07	NCAPG	Non-SMC condensin I complex, subunit G	
		A_70_P037556	−2.12	1.01	NDC80	NDC80 homolog, kinetochore complex component (S. cerevisiae)	
		A_70_P034256	−2.20	1.05	TFDP2	Transcription factor Dp-2 (E2F dimerization partner 2)	
		A_70_P061801	−2.01	−1.39	UBE2C	Ubiquitin-conjugating enzyme E2C	
Condensed chromosome	1.71E-03	CUST_7895_PI375351158	−2.79	−1.15	HMGB1	High-mobility group box 1	
		A_70_P003816	−2.20	−1.67	TEX12	Testis expressed 12	
Alternative splicing	2.04E-03	A_70_P059171	−2.57	−2.17	CLTA	Clathrin, light chain (Lca)	
		A_70_P033441	1.13	2.27	MEST	Mesoderm specific transcript homolog (mouse)	
		A_70_P039536	−2.86	−1.34	MX1	Myxovirus (influenza virus) resistance 1, interferon-inducible protein p78 (mouse)	
		A_70_P060791	2.25	1.38	PEG3	Paternally expressed 3	
		A_70_P015446	−1.52	−2.13	UNC50	Unc-50 homolog (C. elegans)	
Chromosome	2.20E-03	A_70_P065046	−2.68	−1.32	JRKL	Jerky homolog-like (mouse)	
		A_70_P040916	−2.62	−1.67	ORC1L	Origin recognition complex, subunit 1-like (yeast)	
		CUST_12205_PI375351158	−2.04	−1.20	TOP2A	Topoisomerase (DNA) II alpha 170 kDa	[33]
Extracellular matrix	3.15E-03	A_70_P021711	−2.19	−1.69	COL1A1	Collagen, type I, alpha 1	[10,16]
		CUST_11292_PI375351158	2.40	1.12	CRISPLD2	Cysteine-rich secretory protein LCCL domain containing 2	
		A_70_P007356	2.20	1.26	DCN	Decorin	[10,16]
		CUST_7449_PI375351158	1.46	−2.05	DPT	Dermatopontin	
		A_70_P032476	2.45	−1.35	MFAP5	Microfibrillar associated protein 5	[10]
		A_70_P058626	2.27	1.13	VWF	Von Willebrand factor	
Signal peptide	3.25E-03	A_70_P064081	2.44	−2.18	CA4	Carbonic anhydrase IV	
		A_70_P061221	1.20	−2.43	P4HA1	Prolyl 4-hydroxylase, alpha polypeptide I	[10,11]
		A_70_P040831	−2.09	−1.07	SELL	Selectin L	
Tumor suppressor	1.47E-02	A_70_P035971	2.05	−1.75	VWA5A	Von Willebrand factor A domain containing 5A	
		A_70_P026737	3.11	−1.41	WT1	Wilms tumor 1	
		A_70_P061691	1.26	2.26	HPGD	Hydroxyprostaglandin dehydrogenase 15-(NAD)	
Cytoplasm	1.82E-02	A_70_P016337	−1.78	−2.72	HMMR	Hyaluronan-mediated motility receptor (RHAMM)	
		CUST_12176_PI375351158	−1.04	−2.92	ACTG2	Actin, gamma 2, smooth muscle, enteric	
		A_70_P019656	1.01	3.59	ADH1A	Alcohol dehydrogenase 1A (class I), alpha polypeptide	
		A_70_P045301	3.70	2.33	BBOX1*	Butyrobetaine (gamma), 2-oxoglutarate dioxygenase (gamma-butyrobetaine hydroxylase) 1	
		A_70_P069971	−2.09	−1.73	CDC42EP5	CDC42 effector protein (Rho GTPase binding) 5	
		CUST_5823_PI375351158	1.12	2.07	NIN	Ninein (GSK3B interacting protein)	
		A_70_P018021	−3.25	−1.15	PAIP2	Poly(A) binding protein interacting protein 2	
		A_70_P054126	−2.16	−1.11	PHAX	Phosphorylated adaptor for RNA export	
		A_70_P044246	1.26	−2.14	RRAGD	Ras-related GTP binding D	
		A_70_P055106	2.05	1.07	SORBS2	Sorbin and SH3 domain containing 2	
		A_70_P025726	−2.39	−1.60	STMN2	Stathmin-like 2	
		A_70_P060991	−2.80	1.21	CA3	Carbonic anhydrase III, muscle specific	
Fatty acid metabolism	2.26E-02	CUST_8730_PI375351158	1.06	−2.05	ACSL4	Acyl-CoA synthetase long-chain family member 4	
		A_70_P039986	−1.27	2.39	SLC27A2	Solute carrier family 27 (fatty acid transporter), member 2	
Phosphatidic acid metabolic process	2.86E-02	A_70_P039726	−2.50	−1.10	AGPAT1	1-acylglycerol-3-phosphate O-acyltransferase 1 (lysophosphatidic acid acyltransferase, alpha)	
		A_70_P025121	−3.34	−1.30	PLA2G2A	Phospholipase A2, group IIA (platelets, synovial fluid)	
Protein dimerization activity	2.91E-02	CUST_206_PI396851701	2.04	−2.28	FOS	v-fos FBJ murine osteosarcoma viral oncogene homolog	[10,11,33]
		CUST_10535_PI375351158	−2.41	−2.41	NFE2L1	Nuclear factor (erythroid-derived 2)-like 1	
Zinc finger, C2H2-type	3.09E-02	CUST_13307_PI375351158	−2.34	−1.26	ATMIN	ATM interactor	
		A_70_P036056	−2.65	−1.71	KLF6	Kruppel-like factor 6	
		A_70_P065371	−2.27	−1.13	ZCCHC6	Zinc finger, CCHC domain containing 6	
		A_70_P014286	1.33	2.01	ZNF45	Zinc finger protein 45	
Cytoplasmic membrane-bounded vesicle	3.21E-02	A_70_P062831	−3.71	−1.48	EHD3	EH-domain containing 3	
		A_70_P044621	2.27	−1.04	MALL	Mal, T-cell differentiation protein-like	
Metal ion transport	3.39E-02	A_70_P030126	−2.36	1.24	CP*	Ceruloplasmin (ferroxidase)	[10,16]
		CUST_12632_PI375351158	2.28	1.50	KCTD12	Potassium channel tetramerisation domain containing 12	
		A_70_P027206	2.15	2.10	SLC30A1	Solute carrier family 30 (zinc transporter), member 1	[10,11]
		A_70_P050456	−2.65	−1.58	SLC38A10	Solute carrier family 38, member 10	
Antimicrobial	5.05E-02	CUST_139_PI396851701	15.26	−1.74	CATHL1	Cathelicidin 1	
		CUST_3993_PI375351158	5.96	−1.25	S100A8	S100 calcium binding protein A8	[10]
Apoptosis	5.45E-02	CUST_260_PI396851701	−2.04	−1.55	CARD6	Caspase recruitment domain family, member 6	
		A_70_P022526	−2.29	−1.16	OPA1	Optic atrophy 1 (autosomal dominant)	
		A_70_P049526	−1.12	−2.70	PERP	PERP, TP53 apoptosis effector	
		A_70_P069836	−3.14	−1.31	RNF130	Ring finger protein 130	
		A_70_P067091	−2.90	−1.55	UBE2Z	Ubiquitin-conjugating enzyme E2Z	
Immunoglobulin-like fold	5.55E-02	A_70_P036361	2.06	2.09	CD1E	CD1e molecule	
		CUST_9656_PI375351158	−2.03	1.11	FGFR2	Fibroblast growth factor receptor 2	
		CUST_12141_PI375351158	2.64	−1.00	IGK	Ig kappa chain	
		A_70_P016986	−2.23	1.16	TRD@	T cell receptor delta locus	[11]
Secreted	5.70E-02	A_70_P048761	1.34	−2.27	SPINK5	Serine peptidase inhibitor, Kazal type 5	
		CUST_9580_PI375351158	1.13	−2.49	MMRN1	Multimerin 1	
		CUST_10101_PI375351158	−1.07	−2.53	NPNT	Nephronectin	
		A_70_P034356	2.88	1.19	SEMA3G	Sema domain, immunoglobulin domain (Ig), short basic domain, secreted, (semaphorin) 3G	
		A_70_P063891	2.38	−1.22	PCOLCE2	Procollagen C-endopeptidase enhancer 2	
GTP binding	7.49E-02	A_70_P019676	4.33	1.16	ACSM1	Acyl-CoA synthetase medium-chain family member 1	[10]
		A_70_P046951	−2.24	−1.02	ARL4C	ADP-ribosylation factor-like 4C	
		A_70_P008796	−2.06	1.15	DOCK11	Dedicator of cytokinesis 11	
		A_70_P000126	−3.63	−1.82	GBP4	Guanylate binding protein 4	
Disulfide bond	7.58E-02	A_70_P018376	2.56	−1.08	FAP	Fibroblast activation protein, alpha	
		A_70_P041321	−2.15	−2.57	IGHA2	Immunoglobulin heavy constant alpha 2 (A2m marker)	
Structural molecule activity	8.04E-02	A_70_P028306	−2.03	−1.05	LMNB1	Lamin B1	
		CUST_13373_PI375351158	−2.15	−1.29	LOC531679	Ribosomal protein 17-like; similar to 60S ribosomal Protein L17 (L23); similar to ribosomal protein L17; similar to Rpl17 protein; similar to mCG3798	
		CUST_7057_PI375351158	−2.33	−1.07	LOC789587	Similar to Ribosomal_L22 domain containing protein RGD1359290	
		A_70_P031441	−2.43	−1.79	VCL	Vinculin	[11]
Positive regulation of inflammatory response	9.48E-02	A_70_P058551	8.25	−1.12	FABP4	Fatty acid binding protein 4, adipocyte	
		CUST_9779_PI375351158	−1.40	−2.38	IDO1	Indoleamine 2,3-dioxygenase 1	
Other		CUST_13467_PI375351158	1.29	−2.14	FBP2	Fructose-1,6-bisphosphatase 2	
		A_70_P055306	2.77	1.50	SLC43A1	Solute carrier family 43, member 1	
		A_70_P049406	−1.99	−2.32	EIF5	Eukaryotic translation initiation factor 5	[11]
		A_70_P070931	−2.87	−1.18	NUDT19	Nudix (nucleoside diphosphate linked moiety X)-type motif 19	
		A_70_P036101	1.22	−3.18	NOP10	NOP10 ribonucleoprotein homolog (yeast)	[11]
		A_70_P011091	−2.74	−2.01	PFDN2*	Prefoldin subunit 2	
		A_70_P039656	17.20	−1.52	BAC5	5 kDa bactinecin precursor	
		A_70_P061341	−1.03	−2.94	C11ORF10	Chromosome 11 open reading frame 10	
		A_70_P061266	2.36	1.03	C5ORF43	Chromosome 5 open reading frame 43	
		A_70_P051377	14.99	−1.48	CATHL1B	Procyclic dodecapeptide	
		A_70_P015101	−2.70	−1.03	CCDC82	Coiled-coil domain containing 82	
		A_70_P012541	−1.02	−2.36	CNN1	Calponin 1, basic, smooth muscle	
		A_70_P002841	2.03	1.48	EPAS	Endothelial PAS domain protein 1	
		A_70_P008841	−2.18	−1.23	EPSTI1	Epithelial stromal interaction 1 (breast)	
		A_70_P060756	−2.12	1.08	FAM190B	KIAA1128	
		A_70_P062951	2.05	1.12	FNDC3B	Fibronectin type III domain containing 3B	[33]
		A_70_P051181	2.76	1.28	HSD11K	NAD-dependent 11-beta-hydroxysteroid dehydrogenase	
		CUST_46_PI396851701	−2.06	−2.15	IGHA	Immunoglobulin heavy constant alpha	
		A_70_P024671	2.46	1.31	INHBB	Inhibin, beta B	
		A_70_P033656	−2.34	−1.77	ISG12(A)	ISG12(a) protein-like	
		A_70_P012537	−2.19	−3.58	LGALS15	Galectin 15	
		CUST_3620_PI375351158	−1.58	−2.22	LGALS16	Beta-galactoside-binding lectin	
		A_70_P020131	−1.10	−3.84	LGALS9C	Lectin, galactoside-binding, soluble, 9C	
		CUST_6089_PI375351158	−2.02	−1.71	LOC100140018	Similar to cytochrome P450, family 2, subfamily J	
		A_70_P016801	−2.20	−1.48	LOC443321	Lysozyme 2a precursor	
		A_70_P026671	−1.91	−2.05	LOC524810	IgM	
		A_70_P023026	3.09	1.74	LOC615697	Similar to cytochrome P450	
		A_70_P039266	−2.03	−1.83	LOC654331	Pancreatitis-associated protein I	[10]
		A_70_P039271	−3.94	−7.40	MCP-4	Mast cell proteinase-4	
		CUST_11558_PI375351158	2.00	2.23	MGC140754	Hypothetical LOC508613	
		CUST_9725_PI375351158	−2.26	−3.77	PLAC8	Placenta-specific 8	
		CUST_10114_PI375351158	2.56	1.15	RN18S1	18S ribosomal RNA	
		A_70_P030132	−2.40	−1.71	RTP4	Receptor (chemosensory) transporter protein 4	[34]
		A_70_P030426	10.95	1.39	SC5	Cathelin-related prepropeptide	

*Genes selected for validation by quantitative RT-PCR.

BLAST results for clones exhibiting significant alterations in gene expression (*P* ≤ 0.05 and ≥ 2-fold change) in preclinical and clinical stages of natural scrapie. The GO was determined using DAVID Bioinformatics Resources 6.7 (NIAID/NIH, USA), an on-line functional annotation tool. Only genes with a known GO are shown. (C, Clinical; FC, Fold change; NC, Negative control; PC, Preclinical). Previous studies describing altered expression of the differentially expressed genes reported here in the brain and lymphoid tissues of experimentally-infected sheep are cited.

**Table 3:** Signaling pathways associated with differentially expressed genes

**Term**	***P *** **value**	**ProbeName**	**FC (C vs NC)**	**FC (PC vs NC)**	**Gene symbol**	**Gene name**
PPAR signaling pathway	1.88E-02	CUST_598_PI375351158	2.28	1.33	FABP5	Fatty acid binding protein 5 (psoriasis-associated)
		CUST_8730_PI375351158	1.06	−2.05	ACSL4	Acyl-CoA synthetase long-chain family member 4
		A_70_P058551	8.25	−1.12	FABP4	Fatty acid binding protein 4, adipocyte
		A_70_P039986	−1.27	2.39	SLC27A2	Solute carrier family 27 (fatty acid transporter), member 2
ECM-receptor interaction	2.53E-02	A_70_P021711	−2.19	−1.69	COL1A1	Collagen, type I, alpha 1
		A_70_P058626	2.27	1.13	VWF	von Willebrand factor
		A_70_P016337	−1.78	−2.72	HMMR	Hyaluronan-mediated motility receptor (RHAMM)
Focal adhesion	6.73E-02	A_70_P021711	−2.19	−1.69	COL1A1	Collagen, type I, alpha 1
		A_70_P058626	2.27	1.13	VWF	von Willebrand factor
		A_70_P050922	2.19	−1.06	VEGFA	Vascular endothelial growth factor A
		A_70_P031441	−2.43	−1.79	VCL	Vinculin

Signaling pathways with which differentially expressed genes (*P* ≤ 0.05 and ≥ 2-fold change) are associated were identified by enrichment analysis using the on-line functional annotation (DAVID Bioinformatics Resources 6.7; NIAID/NIH, USA). (C, Clinical; FC, Fold change; NC, Negative control; PC, Preclinical).

The relatively low number of animals analyzed and the fact that they were obtained from different geographical locations may limit the statistical power of our study. However, it is difficult to perform this kind of study with a larger number of animals due to the paucity of natural scrapie cases. Moreover, finding negative age-matched controls with the same *PRNP* genotype within the same scrapie-affected flocks is not possible since the disease attack-rate in these animals is close to 100%.

### Validation of gene expression profiling by quantitative RT-PCR

**Figure 3:**
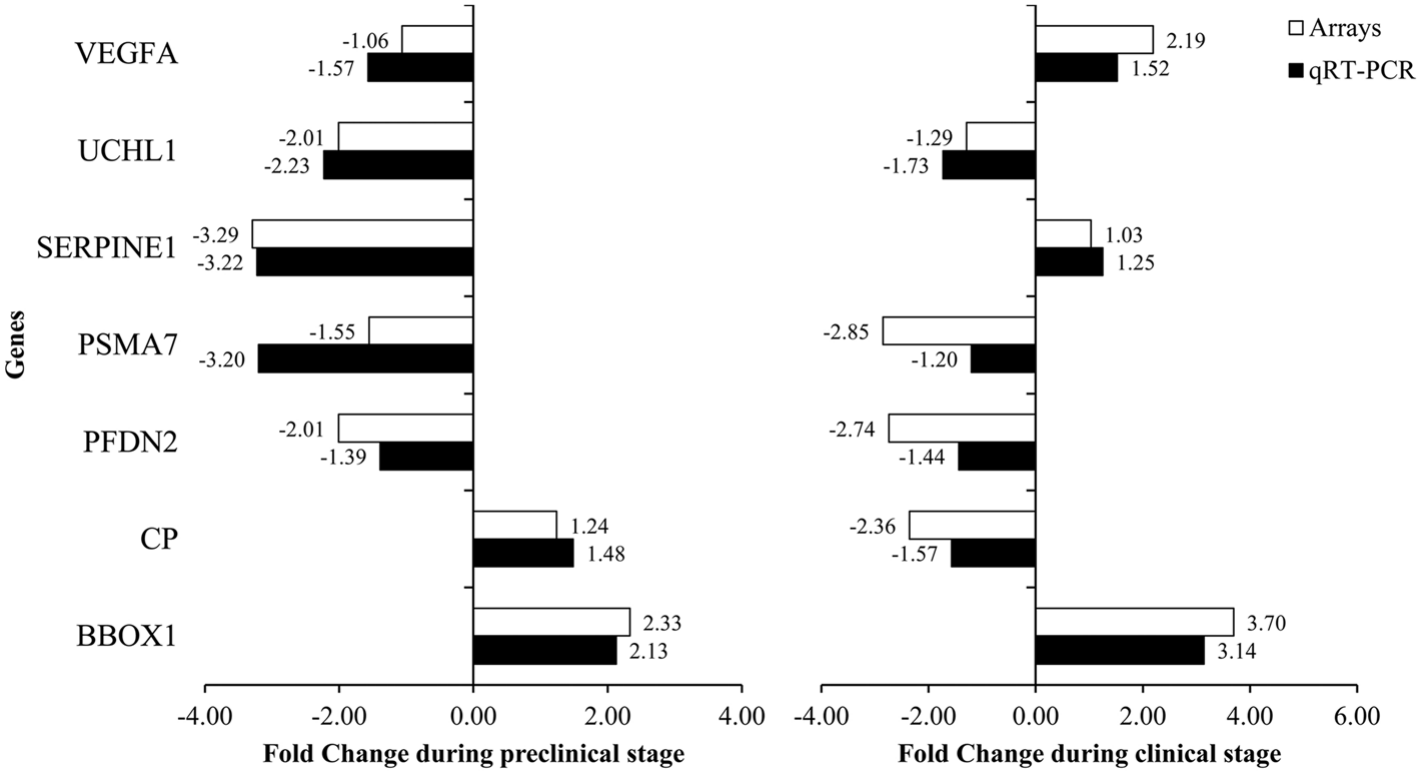
**Confirmation of microarray results by quantitative real-time PCR.** Differential expression of selected genes analyzed by microarray and quantitative RT-PCR in mesenteric lymph node samples from preclinical and clinical scrapie sheep: gamma-butyrobetaine hydroxylase (*BBOX1*), ceruloplasmin (*CP*), prefoldin subunit 2 (*PFDN2*), proteasome subunit alpha type-7 (*PSMA7*), plasminogen activator inhibitor-1 (*SERPINE1*), ubiquitin carboxy-terminal hydrolase L1 (*UCHL1*), and vascular endothelial growth factor (*VEGFA*).

To confirm the microarray results, the expression of 7 genes (*BBOX1*, *CP*, *PFDN2*, *PSMA7*, *SERPINE1*, *UCHL1* and *VEGFA*) was analyzed by qRT-PCR. The expression of 4 of the selected targets (*BBOX1*, *PFDN2*, *PSMA7* and *UCHL1*) was altered in the same direction in both the clinical and preclinical stages of scrapie disease. Expression levels of the other 3 genes (*CP*, *SERPINE1* and *VEGFA*) were altered in opposite directions in the clinical and preclinical stages, respectively. In all cases, qRT-PCR confirmed the changes observed in the microarray hybridizations (Figure 3), revealing statistically significant differences between control and scrapie groups.

### Identification of prion deposition-related genes

**Figure 4:**
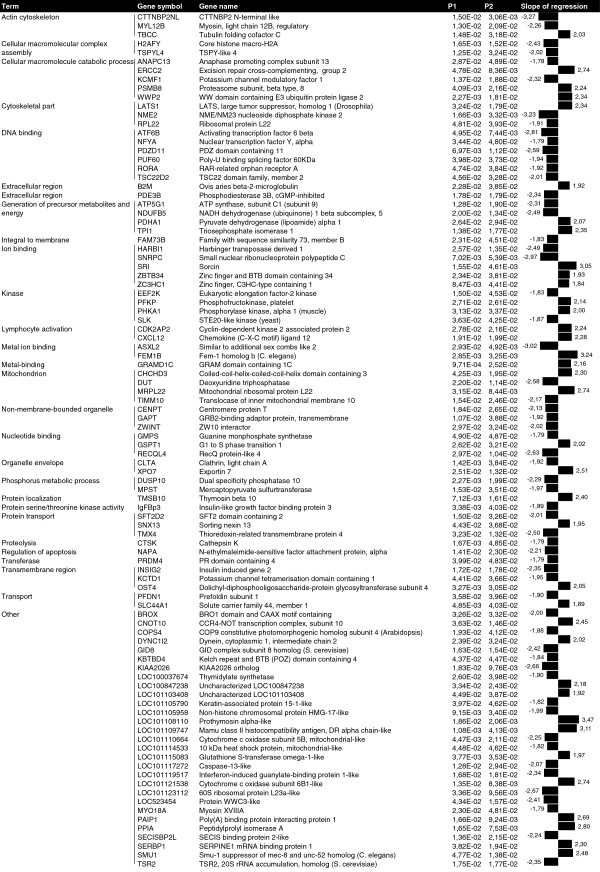
**Relationship between gene expression profiles and PrP**^**Sc **^**deposition.** Figure shows genes whose expression was statistically associated with PrP^Sc^ deposition. P1 is the probability calculated using the simple regression model *y*_*i*_ = *μ* + *bp*_*i*_ + *e*_*i*_, and P2 is the probability calculated using the second regression model *y*_*ij*_ = *μ* + *T*_*i*_ + *bp*_*ij*_ + *e*_*ij*_ (which includes a systematic effect associated with the 3 categories; Preclinical, Clinical and Healthy). The slope of regression describing the relationship between histopathological lesions and gene expression was calculated using the second model. Analyses were performed using R software (R project for Statistical Computing).

Two regression models were used to identify genes associated with prion deposition. One is a regression model that relates gene expression with prion deposition scores regardless of group (Preclinical, Clinical or Healthy), while the other includes a systematic effect associated with the 3 different animal categories and analyzes the remaining variation after subtracting the variability associated with each of the 3 groups. Natural conditions normally display high variability within groups. Using these regression analyses we identified genes whose expression was associated with prion deposition, even if the changes in expression were not significant. We identified 96 genes with known functions whose expression was significantly associated with PrP^Sc^ deposition in both regression models (Figure 4). The slope value represented was obtained using the second regression model. The gene ontology analysis revealed that genes associated with prion deposition were involved in DNA binding, the generation of precursor metabolites and energy, ion binding, catabolic process and kinase functions. Only statistically significant slopes (*p* < 0.05) for both association models are presented in Figure 4.

## Discussion

Mesenteric lymph nodes are one of the first organs in which PrP^Sc^ accumulates [5]. To the best of our knowledge, ours is the first study to use gene expression microarray technology to analyze mesenteric lymph nodes from naturally-infected sheep during the preclinical and clinical stages of scrapie.

Our microarray hybridization analysis identified 139 annotated genes of known functions that exhibited changes in expression of 2-fold or more with respect to controls during at least one of the 2 disease stages analyzed. Of these 139 genes, 105 were differentially regulated in clinical scrapie-infected sheep and 50 in preclinical scrapie-infected sheep. These findings indicate that in the early disease stages, fewer genes are activated or the changes in expression are not sufficiently large to be detected by microarray. PrP^Sc^ accumulation in MLN peaked during the preclinical phase of scrapie (Figure 1), indicating that dysregulated genes that were not common to both disease stages could not be specifically linked to the prion accumulation process.

Prion infection of neural cell lines has revealed a limited transcriptional response and clear differences in transcriptional profiles between different cell lines and between cell cultures and whole brains from infected mice [35-37]. Similarly, our results differ from the profiles described in the brains of sheep with natural scrapie [10,11], indicating the absence of universal changes associated with prion infection.

The differentially expressed genes identified in the present study encode proteins located in almost all subcellular locations: membranes (glycoproteins), the extracellular matrix, the cytoplasm and chromosomes. These proteins participate in general cell processes such as the cell cycle, apoptosis, alternative splicing and chromosome condensation. Genes associated with glycoprotein metabolism, the extracellular matrix and the cell cycle have been previously detected in leukocyte-depleted splenic cells from mice infected with scrapie [16]. We found that gene encoding decorin (DCN), a protein located in the extracellular matrix, was overexpressed in scrapie animals, in line with previous reports of DCN upregulation at both the gene and protein level in cells isolated from scrapie-infected mouse spleen [16]. Our results confirm the differential regulation of *DCN* in the MLN of sheep with natural scrapie. Some of differentially transcribed genes that we identified encode proteins that may be involved in the pathogenesis of protein misfolding diseases. Examples include proteins involved in disulfide bonding, GTP binding and protein dimerization. Differential expression of some of these genes has been reported in other tissues in scrapie-infected mice [12,14,38] and in natural scrapie [10,11].

The GO enrichment analysis of our microarray data identified 3 signaling pathways that may be involved in the pathogenesis of scrapie; extracellular matrix (ECM)-receptor interaction, focal adhesion and the peroxisome proliferator-activated receptor (PPAR) signaling pathway. We and others have previously reported altered expression of *COL1A2*, *COL3A1* and *COL12A1* and other ECM related genes in the CNS of sheep infected with scrapie and other prion diseases [10,11,39,40] and in leukocyte-depleted splenic cells from scrapie-infected mice [16]. Moreover, prion protein regulates β1 integrin signaling activity in prion-infected neurons [41] and the expression of genes of the PPAR signaling pathway is altered in Alzheimer’s disease (AD) [42,43]. Our findings indicate alterations in the extracellular matrix, focal adhesion and in fatty acid metabolism via the PPAR signaling pathway in non-neural tissues susceptible to prion infection.

In addition to analyzing differential gene expression, we investigated the effect of prion deposition on LRS gene expression in order to further our understanding of the prion-specific pathogenic pathways in scrapie. Two regression models (see Methods section) were used to identify possible associations between gene expression data and the PrP^Sc^ deposition score, as determined by immunohistochemistry. These statistical association approaches can identify genes that may be implicated in prion-specific processes, although the differences in their expression levels between control and infected animals were not significant. Except for *CLTA*, none of the genes identified in the association analysis exhibited significant differences in expression in the array data. The gene ontology analysis revealed that genes associated with prion deposition are involved in DNA binding, the generation of precursor metabolites and energy, ion binding, catabolic processes and kinase functions. Among the genes that were closely associated with prion protein deposition were cathepsin K (*CTSK*) and clathrin, light chain A (*CLTA*). Several members of the cathepsin family (including cathepsins C, D, H, S and Z) are differentially expressed in prion-affected brain tissues [12,14,38,44,45]. We observed a negative association between *CTSK* expression and prion deposition, suggesting a possible proteolytic effect of cathepsin K on prion protein. Furthermore, *CLTA* expression was negatively associated with prion deposition. The expression of this gene is also altered in many neurodegenerative diseases such as Alzheimer’s and Huntington’s diseases [46,47], and clathrin-mediated pathways are intimately involved in amyloid formation in neurodegenerative diseases [48]. These observations suggest that *CLTA* may also be involved in prion amyloid formation.

Seven of the differentially expressed genes detected were selected for further analysis by qRT-PCR. These genes were chosen based on their involvement in protein misfolding pathways (*PFDN2*, *UCHL1* and *PSMA7*), the regulation of angiogenesis (*VEGFA* and *SERPINE1*), iron metabolism and neurotoxicity (*CP*) and antioxidant mechanisms (*BBOX1*). Comparable fold changes in the expression of all 7 genes, in both preclinical and clinical disease stages, were observed in the microarray and qRT-PCR analyses, confirming the validity of both the array data and the alignment analysis.

Here, we will briefly discuss how these genes may be involved in PrP^Sc^ formation, dissemination and toxicity and in the response of the organism to prion infection. Genes encoding proteins involved in molecular pathways that regulate protein misfolding (*PSMA7*, *UCHL1* and *PFDN2*) were downregulated in scrapie-infected animals. Furthermore, our association study revealed a positive association between proteasome subunit, beta type, 8 (*PSMB8*) and PrP^Sc^ deposition. The proteins encoded by *PSMA7*, *UCHL1* and *PSMB8* form part of the ubiquitin proteasome system (UPS), a complex that regulates the degradation of incomplete, damaged or misfolded proteins [49,50]. The UCHL1 protein has been implicated in protein misfolding diseases. UCHL1 is abundantly expressed in nervous system tissues [51], and gene variants and changes in the activity of UCHL1 have been associated with neurodegenerative disorders such as Alzheimer’s, Parkinson’s and Huntington’s diseases [27,52-55]. In prion diseases, the functional capacity of the UPS is impaired by the direct interaction of β-sheet-rich PrP with the 20S core particle of the proteasome complex, inhibiting substrate entry [56]. UPS impairment may enable the conversion of cytosolic PrP^C^ to an abnormal, “PrP^Sc^-like” form [57]. Increased levels of ubiquitin conjugates are found in scrapie-infected mouse brains, and are correlated with decreased proteasome function [58]. The downregulation of *PSMA7* and *UCHL1* genes and the positive association of PrP^Sc^ deposition with *PSMB8* expression in both preclinical and clinical stages of natural scrapie in the LRS of sheep is in line with the impaired proteasome function described in prion diseases of the nervous system [28]. Moreover, prefoldin (PFD) is likely to bind to substrate proteins that exist in an unfolded state and to transfer these proteins to the cytosolic chaperonin-containing TCP-1 (CCT) for functional folding [59]. The role of this protein in protein misfolding diseases has been investigated and prefoldin subunit 2 (PFDN2) has been implicated in AD [60]. Our findings reveal downregulation of *PFDN2* expression in both preclinical and clinical stages of natural scrapie in the LRS of sheep. The observed downregulation of *PSMA7* and *UCHL1* may facilitate prion conversion, implicating these genes in the pathogenic mechanism of the scrapie.

The expression of *SERPINE 1* or plasminogen activator inhibitor-1 (PAI-1) was downregulated in preclinical scrapie animals and upregulated in the clinical phase of the disease, suggesting a dual role for this gene in scrapie. SERPINE1 is an inhibitor of 2 types of plasminogen activators; tissue-type plasminogen (tPA) and urokinase-type plasminogen (uPA) activators. Plasminogen was recently show to stimulate PrP misfolding by accelerating the conversion of PrP^C^ to PrP^Sc^[26]. The downregulation of SERPINE1 in preclinical scrapie may inhibit PrP^Sc^ formation in the early stages of the disease. SERPINE1 is also involved in angiogenesis, as evidenced by the defective angiogenesis seen in tumor-bearing SERPINE1-deficient mice [61]. Accordingly, decreased expression of *SERPINE1* may represent a protective response to PrP^Sc^ propagation during the preclinical stage of natural scrapie. However, we observed a slight increase in *SERPINE1* expression in clinical scrapie-infected sheep as compared with controls (FC = 1.25). The expression of vascular endothelial growth factor (VEGF, also known as VEGFA), which is also involved in angiogenesis, was altered in scrapie. This gene mediates a critical rate-limiting step in physiological angiogenesis and plays a key role in tumor progression and angiogenesis and its expression by primary tumor promotes lymphangiogenesis and facilitates metastasis to sentinel lymph nodes [62]. As observed for *SERPINE1*, we detected a slight decrease in *VEGFA* expression in lymphatic tissues in the preclinical stage of natural scrapie, followed by a moderate increase during the clinical stage of the disease. Lymphatic vessels are one of the proposed routes of prion dissemination [63]. Accordingly, the differential regulation of *SERPINE1* and *VEGFA* observed in scrapie may reflect a role in prion propagation. However, further studies will be necessary to clarify the role of these proteins in prion dissemination.

In addition to genes involved in prion misfolding and propagation, we analyzed the expression of the ceruloplasmin (*CP*) gene, which is involved in neurotoxicity. CP is a ferroxidase that contains 95% of the copper present in blood plasma, and is thus a key mediator of copper transport and metabolism [64]. Copper plays an important role in prion integrity; *in vitro*, copper enhances renaturation and stabilization of PrP^Sc^, restoring protease resistance and infectivity [64]. Increased *CP* expression has been reported in leukocyte-depleted splenic cells from clinical scrapie-infected mice [16]. However, we found that *CP* expression was significantly downregulated in the lymph nodes of scrapie-infected animals in the clinical stage of the disease. In patients with aceruloplasminemia, the predominant clinical symptoms are neurologic and include dystonia, dysarthria, and subcortical dementia due to progressive degeneration of the basal ganglia, as well as selective neuronal loss and increased iron content in the microglia, neurons and basal ganglia [29]. Ceruloplasmin also plays an essential role in normal brain iron metabolism and protects against neuronal loss [65]. Our findings suggest that CP deficiency may contribute to the neuronal degeneration observed in scrapie, although further analyses of brain expression and blood levels of CP will be required to determine its role in scrapie.

We detected upregulation of gamma-butyrobetaine hydroxylase (BBOX1), a gene involved in the response of the organism to oxidative stress. BBOX1 is the enzyme responsible for the biosynthesis of L-carnitine, a molecule involved in fatty acid metabolism with important anti-free radical and antioxidant activities [66]. The acetylated form of L-carnitine (acetyl-L-carnitine) has been proposed as a potential treatment for neurodegenerative diseases, including AD [30]. Our results demonstrate upregulation of *BBOX1* in both clinical and preclinical stages of scrapie in the LRS, perhaps reflecting an increase in the levels of antioxidant molecules (L-carnitine and acetyl-L-carnitine) in response to the damage induced by the prion.

The known functions of the genes that were up- and downregulated in scrapie suggest potential roles in the pathogenesis of prion diseases. However, we cannot rule out the possibility that these changes in expression are a secondary response of the organism to the disease. The findings of this transcriptomic analysis thus need to be complemented by further studies analyzing the cellular localization, protein levels and function of the genes of interest in order to establish their specific roles in prion diseases.

## Conclusions

Our genome-wide expression analysis of the LRS of sheep with natural scrapie, at both preclinical and clinical stages of the disease, has identified new genes that may be involved in the pathogenesis of scrapie. We confirmed the differential expression of 7 genes involved in prion or other neurodegenerative diseases. The downregulation of genes involved in repairing misfolded proteins (*PFDN2*, *PSMA7* and *UCHL1*) may contribute to prion formation. Furthermore, the altered expression of genes that promote angiogenesis in the lymph nodes (*SERPINE1* and *VEGFA*) may facilitate prion dissemination, while the observed decrease in CP expression may contribute to prion toxicity. Our results confirm the altered expression of *BBOX1*, a gene implicated in the systemic response of the organism to prion-induced oxidative stress, in scrapie infected animals. Although further studies are necessary to determine the specific roles of these genes in the pathogenesis of prion diseases, our findings represent an important first step towards identifying candidate genes that may serve as useful biomarkers for the development of early diagnostic tools and therapeutic strategies for scrapie.

### Availability of supporting data

Microarray data are available in the ArrayExpress database: accession number E-MTAB-1346 (http://www.ebi.ac.uk/arrayexpress/experiments/E-MTAB-1346/).

## Abbreviations

ARQ: Alanine arginine glutamine; BBOX1: Gamma-butyrobetaine hydroxylase; BSE: Bovine spongiform encephalopathy; CNS: Central nervous system; CP: Ceruloplasmin; DAB: 3,3′-diaminobenzidine; FDCs: Follicular dendritic cells; G6PDH: Glucose-6-phosphate dehydrogenase; GAPDH: Glyceraldehyde-3-phosphate dehydrogenase; GO: Gene ontology; IHC: Immunohistochemistry; LRS: Lymphoreticular system; MLN: Mesenteric lymph node; PFDN2: Prefoldin subunit 2; PRNP: Prion protein; PrPC: Cellular prion protein; PrPSc: Scrapie prion protein; PSMA7: Proteasome subunit alpha type-7; RIN: RNA integrity number; RPL32: Ribosomal protein l32; SERPINE1: Plasminogen activator inhibitor-1; TBMs: Tingible body macrophages; TSEs: Transmissible spongiform encephalopathies; UCHL1: Carboxy-terminal hydrolase L1; VEGFA: Vascular endothelial growth factor; VRQ: Valine arginine glutamine.

## Competing interests

The authors declare that they have no competing interests.

## Authors’ contributions

HF performed the experiments and drafted the manuscript. IMB helped design the study, participated in the molecular genetic studies and the sequence alignment study, and drafted the manuscript. FH participated in the microarray analysis. LV performed the association analysis. CH participated in the pathological characterization of the animals. DM performed the qRT-PCR experiments. MM helped design the study and drafted the manuscript. AB helped design the microarray and sequence alignment studies. JJB helped design and coordinate the study and to draft the manuscript. RB conceived the study, participated in its design and coordination, and helped to draft the manuscript. All authors read and approved the final version of the manuscript.
